# A Patient-Specific Airway Branching Model for Mechanically Ventilated Patients

**DOI:** 10.1155/2014/645732

**Published:** 2014-08-20

**Authors:** Nor Salwa Damanhuri, Paul D. Docherty, Yeong Shiong Chiew, Erwin J. van Drunen, Thomas Desaive, J. Geoffrey Chase

**Affiliations:** ^1^University of Canterbury, Christchurch 8041, New Zealand; ^2^University of Liège, 4000 Liège, Belgium

## Abstract

*Background*. Respiratory mechanics models have the potential to guide mechanical ventilation. Airway branching models (ABMs) were developed from classical fluid mechanics models but do not provide accurate models of* in vivo* behaviour. Hence, the ABM was improved to include patient-specific parameters and better model observed behaviour (ABMps).* Methods*. The airway pressure drop of the ABMps was compared with the well-accepted dynostatic algorithm (DSA) in patients diagnosed with acute respiratory distress syndrome (ARDS). A scaling factor (*α*) was used to equate the area under the pressure curve (AUC) from the ABMps to the AUC of the DSA and was linked to patient state.* Results*. The ABMps recorded a median *α* value of 0.58 (IQR: 0.54–0.63; range: 0.45–0.66) for these ARDS patients. Significantly lower *α* values were found for individuals with chronic obstructive pulmonary disease (*P* < 0.001).* Conclusion*. The ABMps model allows the estimation of airway pressure drop at each bronchial generation with patient-specific physiological measurements and can be generated from data measured at the bedside. The distribution of patient-specific *α* values indicates that the overall ABM can be readily improved to better match observed data and capture patient condition.

## 1. Introduction

Application of respiratory mechanics metrics and modelling is emerging as a means to guide and improve mechanical ventilation in critical care [1–6]. Patient-specific models enable understanding of individual lung physiology in critically ill patients and are especially important in ARDS. Patient data can be interpreted to generate an* in silico* patient model. Various therapies can be tested on this model and the optimum treatment can be found.

One physiologically relevant model of lung morphology is the airway branching model (ABM) [7, 8]. The ABM defines the human lung as a bifurcating tree with 23 generations and the alveoli are present in all generations beyond approximately generation 17 where gas exchange occurs [9]. The ABM is an idealized model of observed anatomy to which fluid mechanics can be applied. In the ABM, a pressure drop occurs after each branch due to the resistive components of the airway wall and the head loss [10, 11]. By estimating the pressure drop for each of these airway branches, the alveoli pressures can be estimated. This outcome provides the opportunity to monitor “regional” specific alveoli pressures that could be used to prevent overdistension of the lung that could lead to lung injury.

In practice, the ABM has been used to estimate respiratory pressure-flow responses in noncritically ill subjects [7, 8, 12, 13]. However, ABM models are very general, using a set of global airway dimensions that do not reflect patient-specific conditions and have not been validated in critically ill patients with respiratory failure. These issues limit bedside application of this model in monitoring or titrating mechanical ventilation.

The dynostatic algorithm (DSA) is currently the most well-known method to estimate alveoli pressure by producing the dynostatic curve during breathing condition [14, 15]. The estimated alveoli pressure is based on the assumption that airway resistance is always the same during inspiration and expiration at isovolume. However, the ability of this method to guide therapy is limited because it does not provide or include information about airway resistance. It thus cannot offer further insight into patient-specific condition [16, 17]. Furthermore, the DSA is analysed at an assumed quasistatic state, which does not exist in normal ventilation. Therefore, the proposed patient-specific airway branching model (ABMps) seeks to bridge this gap and merge the DSA and ABM models to estimate the pressure drop using the physiological dimensions of human airways.

In particular, this study develops the ABMps to capture patient-specific airway pressure changes and unique patient-specific clinical information that is not available from the general ABM or DSA. Three models are presented: (1) the general ABM; (2) the dynostatic algorithm; and (3) the patient-specific ABMps. These models seek to add the specificity that the DSA lacks while retaining the ability to capture alveolar pressures and thus introduce a mixture of novel elements to the overall modelling approach. The models are compared in a retrospective analysis using clinical data from critically ill mechanical ventilation patients to validate the overall approach. Weibel's model [8] includes alveolar volume. However, this work seeks to capture alveolar pressures and thus does not include alveolar volume, which is a difference in the two analyses.

## 2. Methods

### 2.1. Patient Data and Analysis

In this study, retrospective data from Sundaresan et al. [18] was used to compare the three models. This data was from 10 acute respiratory distress syndrome (ARDS) patients in the Christchurch Hospital Intensive Care Unit (ICU). The patients underwent a modified protocol-based recruitment manoeuvre and mechanically ventilated at different positive end-expiratory pressures (PEEP) of 5, 10, and 15 cmH_2_O using a decreasing inspiratory flow profile [19]. All patients were fully sedated and ventilated using Puritan Bennett PB840 ventilators (Covedib, Boulder, CO, USA) with volume control (tidal volume = 400–600 ml), synchronized intermittent mandatory ventilation (SIMV) mode, throughout the trial. The clinical trials and the use of the data were approved by the New Zealand South Island Regional Ethics Committee. Further details on clinical protocols are reported in work of Chiew et al. [6]. The clinical diagnoses of the patients are shown in Table 1 along with their observed auto-PEEP.

To assess model performance, the area under the pressure drop curve (AUC) for inspiration breathing cycle was measured and compared across the models. AUC was used instead of the sum square error due to its unique ability to also capture the pressure drop trend shape as well as its maximum magnitude. Significance tests were carried out using paired Wilcoxon rank-sum test.

### 2.2. General Airway Branching Model

**Figure 1:**
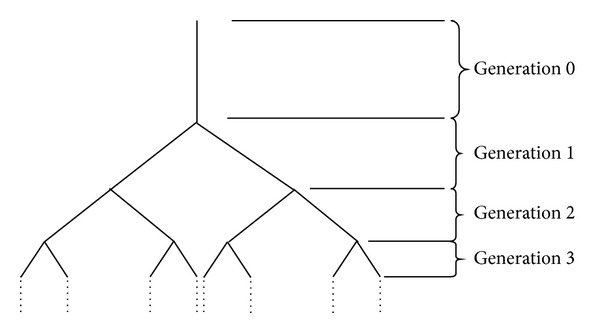
The airway tree structure in which airways are specified by generation number, beginning with trachea [27].

The general ABM is a symmetrical branching tree with physiological airway branching dimensions [20]. Most of the general ABMs assume that the airway generations go up to 23 generations [11, 12]. In this study, the general ABM models the trachea at generation 0 and the alveoli at generations 17–23.  Figure 1 shows the schematic ABM structure and the physical dimensions at every branch generation are shown in Table 2 [12]. It is assumed that the airway dimensions are kept constant during inspiration.

This modelling approach captures head loss as part of Poiseuille model used. Poiseuille flow is defined as

(1) $$\begin{array}{c} \Delta P_{n}=\frac{128\mu LQ}{\pi D^{4}}, \end{array}$$

where *μ* is the dynamic viscosity of air (1.9 × 10^−5^ Pa*·*s /1.9 × 10^−7^ cmH_2_O*·*s),* L* is the length of the particular airway branch,* D* is the diameter of the particular airway branch, and* Q* is the flow rate of airway branch.

Head loss is defined as a pressure drop along the branching system which consists of major and minor losses [13]. The major loss is defined as the pressure drop in the straight section of the airway branching system [13]:

(2) $$\begin{array}{c} \Delta P_{\text{major}}=f\frac{LV^{2}}{2D}. \end{array}$$

The model thus assumes that laminar flow exists in the branches since the diameter for all branches is less than 30 mm with Reynolds number being less than 2000 [10, 11, 21]. Thus, the laminar flow friction factor (*f*) is defined as

(3) f=64Re,

where *Re* is the Reynolds number based on the branch diameter:

(4) Re=ρVDμ,

where *V* is the velocity of the flow of the airway branch. The velocity of the flow can be defined in terms of flow rate:

(5) $$\begin{array}{c} V=\frac{4Q}{{\pi D}^{2}}. \end{array}$$

Hence, substituting (3), (4), and (5) into (2), the major head loss can be derived:

(6) $$\begin{array}{c} P_{\text{major}}=\frac{128\mu LQ}{\pi D^{4}}. \end{array}$$

Equations (1) and (6) show that Poiseuille flow and major loss are the same.

**Figure 2:**
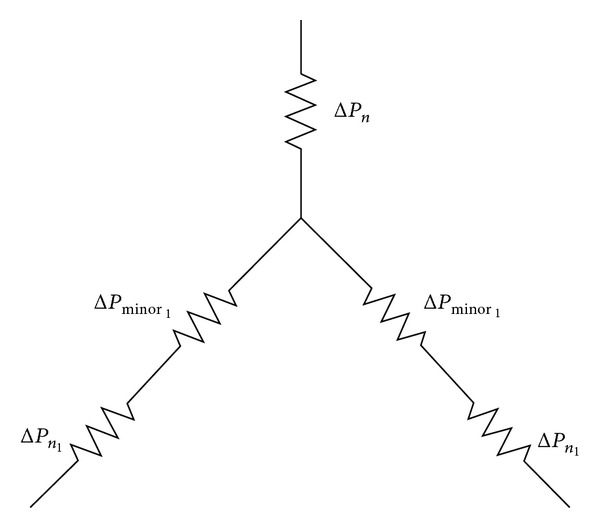
The Poiseuille pressure drop, Δ*P*_*n*_, and minor loss pressure drop, Δ*P*_minor_, at each of the branching respiratory systems for ABMps.

In this specific model, estimates from [20] incorporate minor loss information due to the bifurcation of each branch starting from generation 1, as shown in Figure 2.

Every time the branch bifurcates to the next generation, there is a change in the velocity distribution. Thus, this airway resistance and minor loss will contribute to the pressure drop over the bronchial paths.

In addition to the resistance component of the bronchial part, there is an additional resistance in the endotracheal tube (ETT). All of these patients had ETT with the same dimensions. The length of the ETT was 330 mm and the diameter was 9 mm [22, 23]. The resistance induced by the ETT is added to the overall model results. The ETT is at generation −1 and trachea is at generation 0 and then continues to the remaining generations up to generation 23. With the added ETT in the ABM model, the total pressure drop due to the resistance component, minor loss, and the artificial conducting airway can be modelled as follows:

(7) $$\begin{array}{c} \Delta P_{\text{ABM}}=\Delta P_{n}+\Delta P_{\text{minor}}, \end{array}$$

where

(8) $$\begin{array}{c} \Delta P_{n}=\frac{128\mu }{\pi }\overset{23}{\underset{n=-1}{\sum }}\frac{L_{n}Q_{n}}{2^{n}{D_{n}}^{4}}, \end{array}$$

(9) $$\begin{array}{c} \Delta P_{\text{minor}}=\frac{8K_{L}\rho }{\pi^{2}}\overset{23}{\underset{n=-1}{\sum }}\frac{{Q_{n}}^{2}}{{D_{n}}^{4}}, \end{array}$$

where *K*_*L*_ is the minor loss coefficient (=2) [24],*ρ* is the density (1.25 kg/m^3^), *n* represents the airway branch generation, *L*_*n*_ is the length of the particular airway branch, and *D*_*n*_ is the diameter of the particular airway branch. The flow rate of airway branch (*Q*_*n*_) is assumed to be half of the previous generation flow rate.

This combined model is unique for this clinical application [20]. However, it is entirely general based on the data in Table 2 and fixed structure.

### 2.3. Dynostatic Algorithm Model (DSA)

Another pressure drop estimation is the dynostatic algorithm [15, 25]. Proposed by Kárason et al. [14], it assumes inspiration and expiration airway resistances are the same at isovolume (*R*_insp_ = *R*_exp⁡_ at isovolume). This assumption allows a surrogate of alveolar pressure, known as dynostatic pressure (*P*_dyn_), to be estimated as follows:

(10) $$\begin{array}{c} R_{\text{insp}}=\frac{P_{\text{insp}}-P_{\text{dyn}}}{Q_{\text{insp}}}=R_{\exp}=\frac{P_{\exp}-P_{\text{dyn}}}{Q_{\exp}}, \end{array}$$

where *Q*_exp⁡_ is the expiration flow, *Q*_insp_ is the inspiration flow, *P*_insp_ is the pressure during inspiration, and *P*_exp⁡_ is the pressure during expiration. The pressure drop (Δ*P*_DSA_) during inspiration is estimated as

(11) $$\begin{array}{c} \Delta P_{\text{DSA}}=P_{\text{insp}}-P_{\text{dyn}}, \end{array}$$

where

(12) $$\begin{array}{c} P_{\text{dyn}}=\frac{P_{\text{insp}}\times Q_{\exp}-P_{\exp}\times Q_{\text{insp}}}{Q_{\exp}-Q_{\text{insp}}}. \end{array}$$

**Figure 3:**
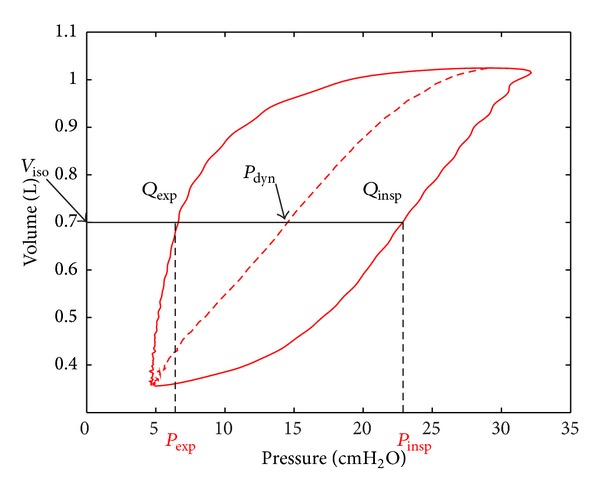
The DSA diagram and resulting quasistatic, single line pressure volume curve.

Figure 3 shows the DSA curve relative to dynamic pressure volume data based on these assumptions. However, this model is strictly static and thus cannot capture any dynamic elements of the observed data, as also seen in Figure 3, and instead approximates the possible underlying static curve. The DSA curve would be obtained if every small increment of pressure was held long enough to achieve a static plateau pressure at each volume increment. While it is useful to estimate the static alveolar pressures, it is not feasible clinically.

### 2.4. Patient-Specific Airway Branching Model (ABMps)

This general ABM presented is extended to account for patient-specific physiological conditions observed in measured pressure and volume data. The ABMps airway pressure drop is defined as

(13) $$\begin{array}{c} \Delta P_{{\text{ABM}}_{\text{PS}}}=\Delta P_{n_{\text{PS}}}+\Delta P_{{\text{minor}}_{\text{PS}}}. \end{array}$$

A patient-specific multiplier (*α*) can be used to uniformly alter the bronchial diameter defined in Table 2 to better match the observed data. Incorporating this factor into (8) and (9) yields the following, respectively:

(14) $$\begin{array}{c} \Delta P_{n_{\text{PS}}}=\frac{128\mu }{\pi }\overset{24}{\underset{n=0}{\sum }}\frac{L_{n}Q_{n}}{2^{n}\alpha^{4}{D_{n}}^{4}}, \end{array}$$

(15) $$\begin{array}{c} \Delta P_{{\text{minor}}_{\text{PS}}}=\frac{8K_{L}\rho }{\pi^{2}}\overset{24}{\underset{n=0}{\sum }}\frac{{Q_{n}}^{2}}{\alpha^{4}{D_{n}}^{4}}, \end{array}$$

where *α* is defined as patient-specific relative of airway diameter and is limited to *α* = [0.45,1.50]. If *α* = 1.0, the patient will follow the general airway dimensions proposed by [7]. If *α* is <1.0, the patient-specific airway is relatively smaller than the Horsfield model [7], perhaps indicating that airway constriction. Finally, if *α* > 1.0, the patient has a larger airway. Larger and smaller airways in this context imply differences in resistance in the observed data. Hence, they may also capture relative overdistension with pressure as well as patient-specific state.

To estimate a patient-specific *α*, Δ*P*_ABM_PS__ is assumed to be the same as Δ*P*_DSA_, where Δ*P*_DSA_ is the most currently well-accepted method to estimate alveoli pressures. Hence, substituting (14) and (15) into (13), a patient-specific *α* can be derived as follows:

(16) $$\begin{array}{c} \alpha =\sqrt[{4}]{\frac{128\mu }{\pi \Delta P_{\text{DSA}}}\overset{24}{\underset{n=0}{\sum }}\frac{L_{n}Q_{n}}{2^{n}{D_{n}}^{4}}+\frac{8K_{L}\rho }{\pi^{2}\Delta P_{\text{DSA}}}\overset{24}{\underset{n=0}{\sum }}\frac{{Q_{n}}^{2}}{{D_{n}}^{4}}}. \end{array}$$

The value for *α* for each patient and PEEP value were calculated using measurements of Δ*P*_DSA_ and *Q*_*n*_ and values of *L*_*n*_ and *D*_*n*_, from Table 2 in (16). Thus, a different *α* value is obtained for each PEEP level and for each patient. The area under the curve (AUC) is the area under the pressure drop curve for all the three models. The AUC of pressure drop with respect to time for a single inspiration cycle shown in Figure 6 provides a good single value measure of the pressure drop when identifying the patient-specific *α*. The AUC of Δ*P*_ABM_PS__ is compared with AUC of Δ*P*_DSA_ for all patients at each PEEP level by calculating the minimum average of the absolute percentage error (APE). This comparison ensures that the model is not overfit to the data but that patient-specific aspects are used to capture and represent the fundamental trend.

## 3. Results

**Figure 4:**
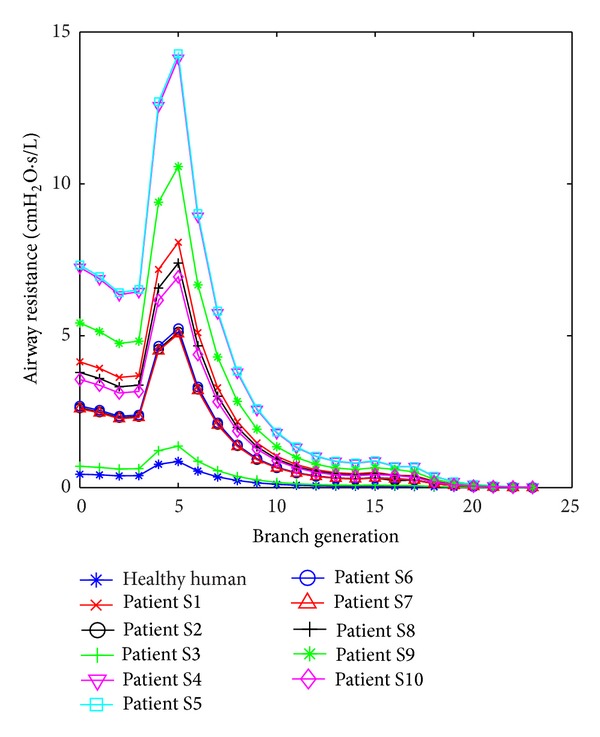
Airway resistance for each branch for every patient of the ABMps model.

The estimated airway resistance for each branch generation is presented in Figure 4. The AUC for all 10 ARDS patients and all 3 models are shown in Table 3. It is clear that the general ABM has a very large difference compared to the DSA (*P* < 0.05).  Table 4 shows patient-specific *α* that relates to the patient disease state. The *α* values for COPD patients were significantly lower than the other patients in the cohort (ranksum *P* < 0.0001, Kolmogorov-Smirnov *P* = 0.001), thus indicating a more resistive airway.

**Figure 5:**
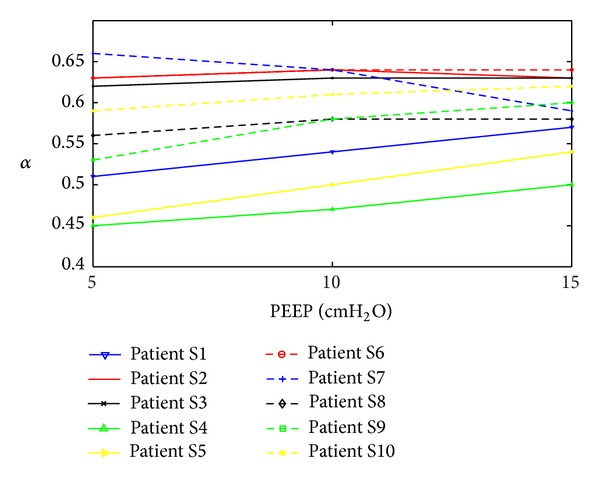
Comparison of all *α* values for all patients versus PEEP for ABMps.

**Figure 6:**
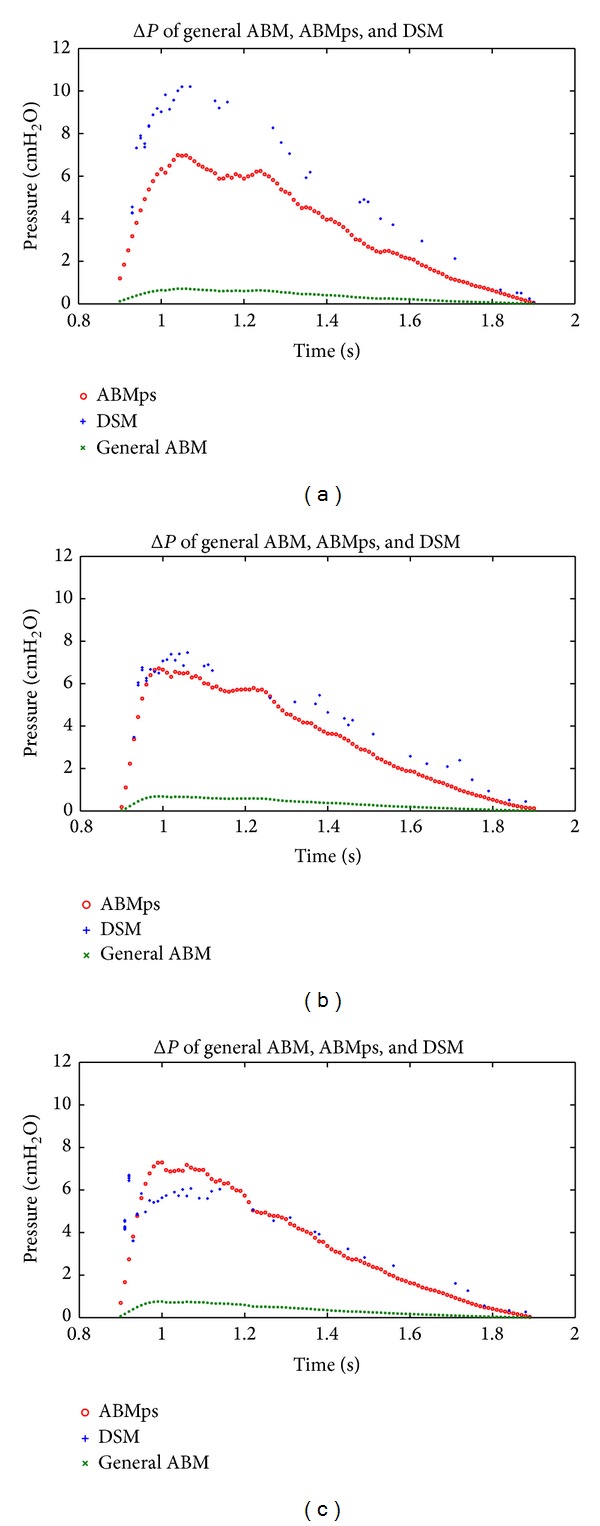
Comparison of airway pressure drop for one breathing cycle for patient S1 with COPD for general ABM, ABMps, and DSA with *α* = 0.57. Plot of general ABM, ABMps, and DSA at (a) PEEP = 5 cmH_2_O, (b) PEEP = 10 cmH_2_O, and (c) PEEP = 15 cmH_2_O.

**Figure 7:**
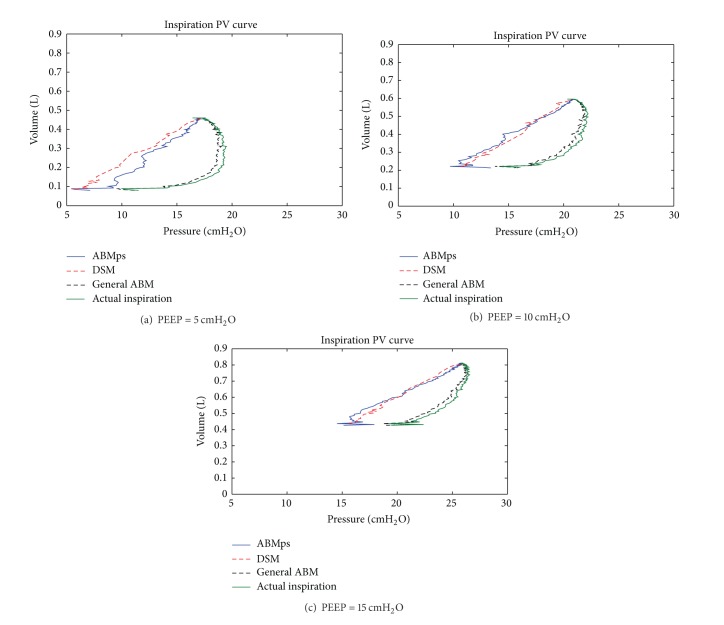
Comparison of pressure volume curve for patient S1 with COPD for general ABM, ABMps, DSA, and actual inspiration with *α* = 0.57.

Figure 5 shows the trend of *α* values for all patients at PEEP = 5 cmH_2_O, 10 cmH_2_O, and 15 cmH_2_O. Figure 6 compares the pressure drop curve for one breathing cycle for patient S1, as an example, for all three models at PEEP = 5 cmH_2_O, 10 cmH_2_O, and 15 cmH_2_O, with *α* = 0.57.  Figure 7 shows the pressure and volume curve for all the three models for the same patient and *α* value in Figure 6.

## 4. Discussion

It can be observed in Figure 4 that the airway resistance is higher at the trachea (generation 0) and 5th generation branch for all patients. Initially, the resistance starts to drop from generation 0, which is the trachea, up to generation 4. The resistance starts to rise at generation 5 as the length of the bronchial tube is higher at this generation compared to the previous branches [12]. Airway obstructions increased the airway resistance, as seen in Figure 4, where, for COPD patients, S1, S4, S5, S9, and S10, the airway resistance was higher compared to the healthy human and other patients. With the increased airway resistance in COPD patients, these results clearly show that a higher resistance results in the higher airway pressure drop observed and thus the consequent reduced volume. This estimation of airway resistance by the ABMps cannot be done by using the DSA model and highlights a useful feature of this approach.

With the patient-specific *α* value, the airway resistance can be estimated, which leads to estimating the pressure drop. Furthermore, the airway resistance for each patient is different and shows that airway resistance is higher for COPD patients. Thus, this ABMps can be used to detect the disease state independently or automatically, which could not be done by the DSA.

The estimated airway pressure drop using the patient-specific ABMps with *α* value was significantly different from the pressure drop estimated using the general ABM (*P* < 0.05). Table 3 shows that AUC pressure drop in the general ABM typically exhibited very large differences for all patients at all PEEP levels compared to the DSA with *P* < 0.05. In contrast, a good comparison is observed between the AUC of pressure drops in ABMps and DSA in Table 3 and Figure 6. This result clearly shows that the general ABM does not capture the observed mechanics of critically ill mechanical ventilation patients despite it being a mix of classical mechanics and measured behaviour [20]. However, if it is extended with patient-specific *α*, it is a far better representation of the patient-specific airway dimension. In addition, these patient-specific aspects dominate the differences from the general ABM modeling approach to matching the patient-specific DSA results. This last result matches the interpatient variability noted in MV patients as a whole and shows the need for a patient-specific approach to estimated model-based alveolar pressures in this cohort.

Figure 6 illustrates an example of estimated pressure drops for patient S1 between general ABM, ABMps, and DSA models. With *α* = 0.57 at PEEP = 15 cmH_2_O, the AUC of pressure drop for patient S1 in ABMps and DSA yields the same result of 3.33 cmH_2_O*·*s, where the general ABM yields a far lower 0.36 cmH_2_O*·*s. This difference indicates that the ABMps was able to predict the same airway pressure drop as DSA by incorporating the *α* term that was unique for each specific patient's branching system. Equally, the general ABM, as defined in Table 2, is not capable of accurately capturing the observed mechanics in mechanical ventilation patients. This difference and small error *α* < 1.0 value are due to the respiratory failure status of these patients.

From Tables 3 and 4, it is also noted that all *α* values for all patients are less than 1.0. Therefore, all pulmonary paths have a smaller diameter than the expected diameters from Table 2. This finding reflects the clinical condition of these patients. In particular, patients with restrictive airway conditions, such as chronic obstructive pulmonary disease (COPD), have constricted airways and respiratory failure by definition. Thus, *α* is smaller comparatively (*α* = 0.45–0.62) than what would be assumed for a healthy individual, as per Table 2. Smaller *α* value also occurred in aspiration patients (*α* = 0.56–0.63) where the restrictive airway condition of the lung is developed due to the entrance of foreign materials into the bronchial generations. Thus, with the use of *α*, ABMps is not only able to capture similar alveolar pressure as DSA but it is also able to track patient disease state over time as shown in Table 4 and Figure 5. The greater airway resistance modelled with *α* < 1.0 results in higher pressure drops at the alveoli, as expected, and is thus a better match with the DSA. In addition, ARDS patients are often associated with regional airway collapse [26] at higher branch generations, which will also greatly alter the airway resistance [12] and supports the overall interpretation presented for these patient-specific results.

Equally, the inspiration pressure volume curve for the general ABM and the ABMps can be modelled and compared with the DSA and the actual dynamic inspiration pressure volume curve, as shown in Figure 7. The general ABM does not capture alveoli pressure like the DSA in critically ill patients. The general ABM would look a lot like the inspiration curve in Figure 7 and shifted slightly to lower pressure. This outcome occurs because the Δ*P* drops are 10 times smaller than the Δ*P* drops in the ABMps in Table 3. Thus, the general ABM was not effective at capturing the estimated alveoli pressure volume curve in this cohort.

Both the ABMps and DSA take into account the airway resistance that occur in the lung and lead to the airway pressure drop. Furthermore, the ABMps is designed with minor loss and the patient-specific airway dimension, *α*, that is unique for each patient. Hence, in Figure 6 the pressure volume curve for the ABMps is very similar to the DSA as PEEP increases from 5 cmH_2_O to 15 cmH_2_O with the patient-specific *α* = 0.57. Although the ABMps had smaller error in comparison to the DSA at lower PEEP, this inspiration pressure volume curve could still be applied as a guidance tool for clinicians to provide a better solution for mechanically ventilated patients.

## 5. Limitations

Although the ABMps estimates pressure drops at every physiological airway branch, there are limitations to its predictive capability. This ABMps assumes that the bifurcations run throughout the entire generations from the 1st generation up to the 23rd generation based on physiological measurements and assumption by referring to Weibel et al. model which has been used widely in deterministic studies [8, 11, 12]. However, this assumption may not be applied in the real scenario if one or more of the bronchial paths are blocked. Nevertheless, the ABMps with the patient specific *α* value is capable of showing that every patient has *α* < 1.0, which reflects that patient's pulmonary paths have a reduced equivalent diameter that results in a different resistance as compared to the healthy human physiological measurements. This reduced equivalent diameter thus is a surrogate that captures the (variable) clinical condition of each patient. For example, COPD patients have a blockage of bronchial portions of the lungs that reduce volume. In contrast, respiratory failure or ARDS patients may experience a similar total loss of lung volume due to collapsed alveoli distributed throughout the lung. However, in both cases, adjustment to MV settings may be needed to try to recruit this lost volume, and, equally, in both cases, additional pressure is the typical mechanism used for this recruitment.

In this research, the ETT dimensions were the same across all patients. However, while ETT dimensions may vary between patients, the ABMps remains capable of estimating this pressure drop so that an accurate estimation of the pressure drop in the deep bronchial paths can be used for predicting the alveolar pressure drop. The ETT dimensions are typically known and were consistent in this research, thus maintaining the ability to estimate this alveolar pressure drop, which is important if used to avoid and prevent further lung injury due to the provision of excessive pressure.

Although an average value of minor loss coefficient is used in this model, the ABMps was able to capture the pressure drop in the airway branching system. In addition, at the very low flows at the later generations, the contributions to pressure drop of these minor loss coefficient constants are almost negligible [13]. Thus, the use of this average value of minor loss coefficient and an ETT specific loss capture much of the loss seen. Equally, while a distribution of loss coefficients based on anatomical studies could be used, it cannot be validated given the limited measurements of pressure and flow available in pulmonary medicine. Thus, given these points, an average value is used because it captures the overall losses and pressure drops, even if intermediate pressures may not be fully accurate, and thus provides a good estimate of alveolar pressure, which is the main goal of this model.

With the use of *α*, ABMps was able to capture similar alveolar pressure as the DSA with further insight of patient-specific airway dimensions during mechanical ventilation. However, due to limited patient data, the application of *α* as a surrogate of patient-specific condition was not fully validated. In particular, as a patient recovers from ARDS, the regional collapsed alveoli may be recruited, resulting in a change in patient airway condition that would be seen in a change in the effective value of *α*. Thus, the clinical utility of patient-specific *α* in tracking patient disease state warrants further investigation over larger cohorts given this initial set of results.

## 6. Conclusions

A patient-specific airway branching model was derived from a general ABM model and was capable of assessing the pressure drop of the airway using clinically available airway pressure and flow measurements. Using this model, the airway condition of a patient can be characterised and thus could provide clinically useful information to clinicians to guide patient-specific therapy. This result shows that even though the ABMps model is based on simple Poiseuille flow and minor loss equations, the extension to a patient-specific airway dimension, *α*, produced consistent trends and compared well with the DSA model as the current standard for estimating alveolar pressures. This *α* value could be calculated at the bedside in a similar fashion to offer additional insight beyond the DSA with respect to potential to recruit volume (increase *α*) and to monitor patient condition. Overall, these results provide a general model framework that can be customised to each patient at the bedside to help guide care. The results justify further prospective trials to assess the clinical utility of patient-specific value of *α* in assessing patient condition.

## Figures and Tables

**Table 1:** Summary of patient auto-PEEP settings [19].

Patient	Sex	Age [years]	Clinical diagnostic	Auto-PEEP [cmH_2_O]
S1	Female	61	Peritonitis, COPD	10
S2	Male	22	Trauma	12
S3	Male	55	Aspiration	10
S4	Male	88	Pneumonia, COPD	10
S5	Male	59	Pneumonia, COPD	12
S6	Male	69	Trauma	11
S7	Male	56	Legionnaires	7.5
S8	Female	45	Aspiration	12
S9	Male	37	H1N1, COPD	12
S10	Male	56	Legionnaires, COPD	3

**Table 2:** Physical measurements of bronchial paths [12].

Branch generations	Diameter (mm)	Length (mm)	Reynolds number
−1 (ETT)	9	330	390
0 (tracheal)	18	120	775
1	12.20	48	573
2	8.30	19	427
3	5.60	8	307
4	4.50	13	198
5–16	3.50–0.60	10.70–1.70	123–0.60
17–22	0.57–0.43	1.50–0.63	0.56–0.41
23	0.40	0.50	0.02

**Table 3:** AUC of airway pressure drops for Sundaresan's patients [18], with PEEP = 5, 10, and 15 cmH_2_O for general ABM, ABM specific, and DSA.

Patient	Auto-PEEP (cmH_2_O)	PEEP (cmH_2_O)	Optimal *α*	ABMps AUC (cmH_2_O*·*s)			General ABM AUC (cmH_2_O*·*s)	DSA AUC (cmH_2_O*·*s)	Error = AUC (ABMps-DSA) (%)
				PEEP 5 cmH_2_O	PEEP 10 cmH_2_O	PEEP 15 cmH_2_O			
S1	10	5	0.51	5.35	5.10	5.18	0.37	5.35	0.14
		10	0.54	4.22	4.02	4.09	0.35	4.02	0.09
		15	0.57	3.44	3.28	3.33	0.36	3.33	0.06

S2	12	5	0.63	3.11	3.03	2.79	0.50	3.11	0.13
		10	0.64	2.99	2.91	2.68	0.49	2.91	0.10
		15	0.63	3.18	3.10	2.86	0.45	2.86	0.19

S3	10	5	0.62	2.89	2.84	2.62	0.42	2.89	0.11
		10	0.63	2.63	2.58	2.38	0.41	2.58	0.08
		15	0.63	2.73	2.68	2.47	0.38	2.47	0.15

S4	10	5	0.45	10.0	9.66	10.30	0.40	10.00	0.21
		10	0.47	8.20	8.05	8.36	0.40	8.05	0.16
		15	0.50	6.59	6.52	6.77	0.41	6.77	0.15

S5	12	5	0.46	8.82	8.45	7.92	0.41	8.82	0.42
		10	0.50	6.76	6.48	6.07	0.39	6.48	0.23
		15	0.54	4.79	4.59	4.30	0.37	4.30	0.26

S6	11	5	0.63	3.03	2.92	2.92	0.46	3.03	0.07
		10	0.64	2.82	2.72	2.72	0.44	2.72	0.04
		15	0.64	2.80	2.70	2.70	0.44	2.70	0.03

S7	7.5	5	0.66	1.80	1.84	1.83	0.34	1.80	0.03
		10	0.64	1.98	2.03	2.02	0.35	2.03	0.02
		15	0.59	2.81	2.88	2.86	0.34	2.86	0.03

S8	12	5	0.56	3.76	3.69	3.71	0.37	3.76	0.04
		10	0.58	3.27	3.20	3.22	0.36	3.20	0.03
		15	0.58	3.09	3.15	3.11	0.36	3.11	0.02

S9	12	5	0.53	5.96	6.02	6.01	0.48	5.96	0.04
		10	0.58	4.42	4.46	4.45	0.49	4.46	0.02
		15	0.60	3.74	3.78	3.77	0.49	3.77	0.01

S10	3	5	0.59	3.76	3.62	3.70	0.47	3.76	0.07
		10	0.61	3.32	3.20	3.27	0.45	3.20	0.06
		15	0.62	3.15	3.04	3.10	0.46	3.10	0.04

Mean			0.58				0.41	4.12	0.07
[IQR]			[0.54–0.63]				[0.37–0.46]	[3.20–4.42]	[0.03–0.15]

**Table 4:** Patient-specific *α* versus disease state.

Number of patients	Clinical diagnostic	Range of *α*
5	COPD	0.45–0.62
2	Aspiration	0.56–0.63
2	Trauma	0.63–0.64
1	Legionnaires	0.59–0.66
